# Supplementary material for: Description and genome-wide analysis of Profundicola chukchiensis gen. nov., sp. nov., marine bacteria isolated from bottom sediments of the Chukchi Sea
Source: PLoS One. 2023 Jul 26;18(7):e0287346. doi: 10.1371/journal.pone.0287346 (PMC10370774; doi:10.1371/journal.pone.0287346)
Supplement: S1 Fig — (DOCX) [file pone.0287346.s001.docx]

**Supplementary materials**

“Description and genome-wide analysis of *Profundicola chukchiensis* gen. nov., sp. nov., marine bacteria isolated from bottom sediments of the Chukchi Sea”

**Lyudmila Romanenko^1^, Nadezhda Otstavnykh^1^, Valeriya Kurilenko^1^, Peter Velansky^2^, Viacheslav Eremeev^1^, Valery Mikhailov^1^, Marina Isaeva^1*^**

^1^G.B. Elyakov Pacific Institute of Bioorganic Chemistry, Far Eastern Branch, Russian Academy of Sciences, Vladivostok, Russia

^2^A.V. Zhirmunsky National Scientific Center of Marine Biology, Far Eastern Branch, Russian Academy of Sciences, Vladivostok, Russia

*****Correspondence: issaeva@gmail.com (MI)

Submitted to PLoS ONE.


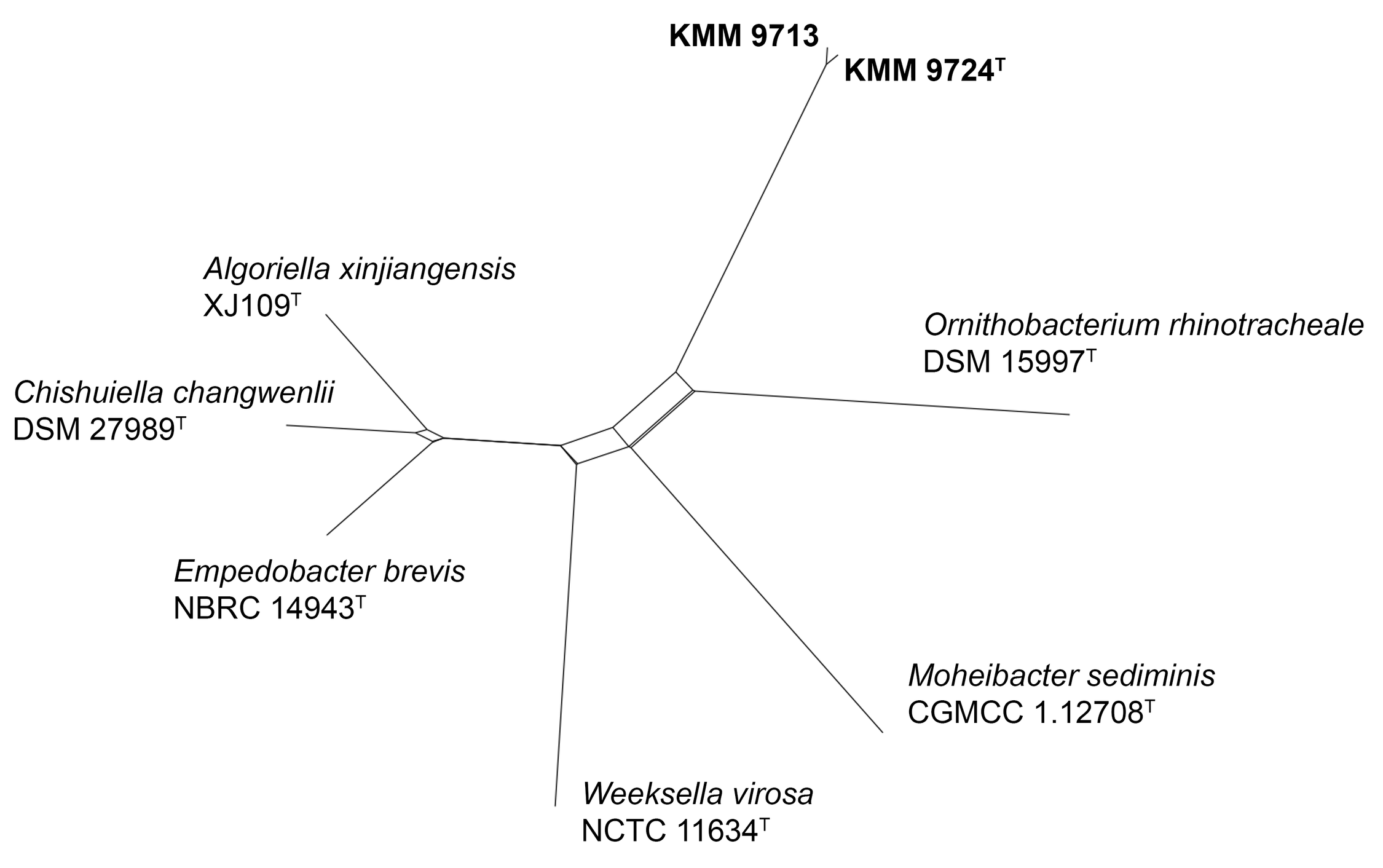


**S1 Fig.** Neighbor-net for novel strains KMM 9724T and KMM 9713 and related members of the family *Weeksellaceae* based on MLSA sequences.

The multilocus sequence analysis (MLSA) was conducted using concatenated sequences of five housekeeping genes, 16S rRNA, *atpD*, *gyrB*, *recA*, and *rpoB*, which were retrieved from whole genome sequences. The sequences obtained were aligned in the MEGA X, version 10.2.1 [1]. Neighbor-net analysis was performed for the concatenated genes using SplitsTree4 version 4.18.2 with a Jukes–Cantor correction [2].

1. Kumar S, Stecher G, Li M, Knyaz C, Tamura K. MEGA X: Molecular evolutionary genetics analysis across computing platforms. Mol. Biol. Evol. 2018; 35: 1547–1549.
2. Huson D H, Bryant D. Application of Phylogenetic Networks in Evolutionary Studies. Mol. Biol. Evol. 2006; 23: 254–267.
