# Supplementary material for: Description and genome-wide analysis of Profundicola chukchiensis gen. nov., sp. nov., marine bacteria isolated from bottom sediments of the Chukchi Sea
Source: PLoS One. 2023 Jul 26;18(7):e0287346. doi: 10.1371/journal.pone.0287346 (PMC10370774; doi:10.1371/journal.pone.0287346)
Supplement: S2 Fig — (DOCX) [file pone.0287346.s002.docx]

**Supplementary materials**

“Description and genome-wide analysis of *Profundicola chukchiensis* gen. nov., sp. nov., marine bacteria isolated from bottom sediments of the Chukchi Sea”

**Lyudmila Romanenko^1^, Nadezhda Otstavnykh^1^, Valeriya Kurilenko^1^, Peter Velansky^2^, Viacheslav Eremeev^1^, Valery Mikhailov^1^, Marina Isaeva^1*^**

^1^G.B. Elyakov Pacific Institute of Bioorganic Chemistry, Far Eastern Branch, Russian Academy of Sciences, Vladivostok, Russia

^2^A.V. Zhirmunsky National Scientific Center of Marine Biology, Far Eastern Branch, Russian Academy of Sciences, Vladivostok, Russia

*****Correspondence: issaeva@gmail.com (MI)

Submitted to PLoS ONE.


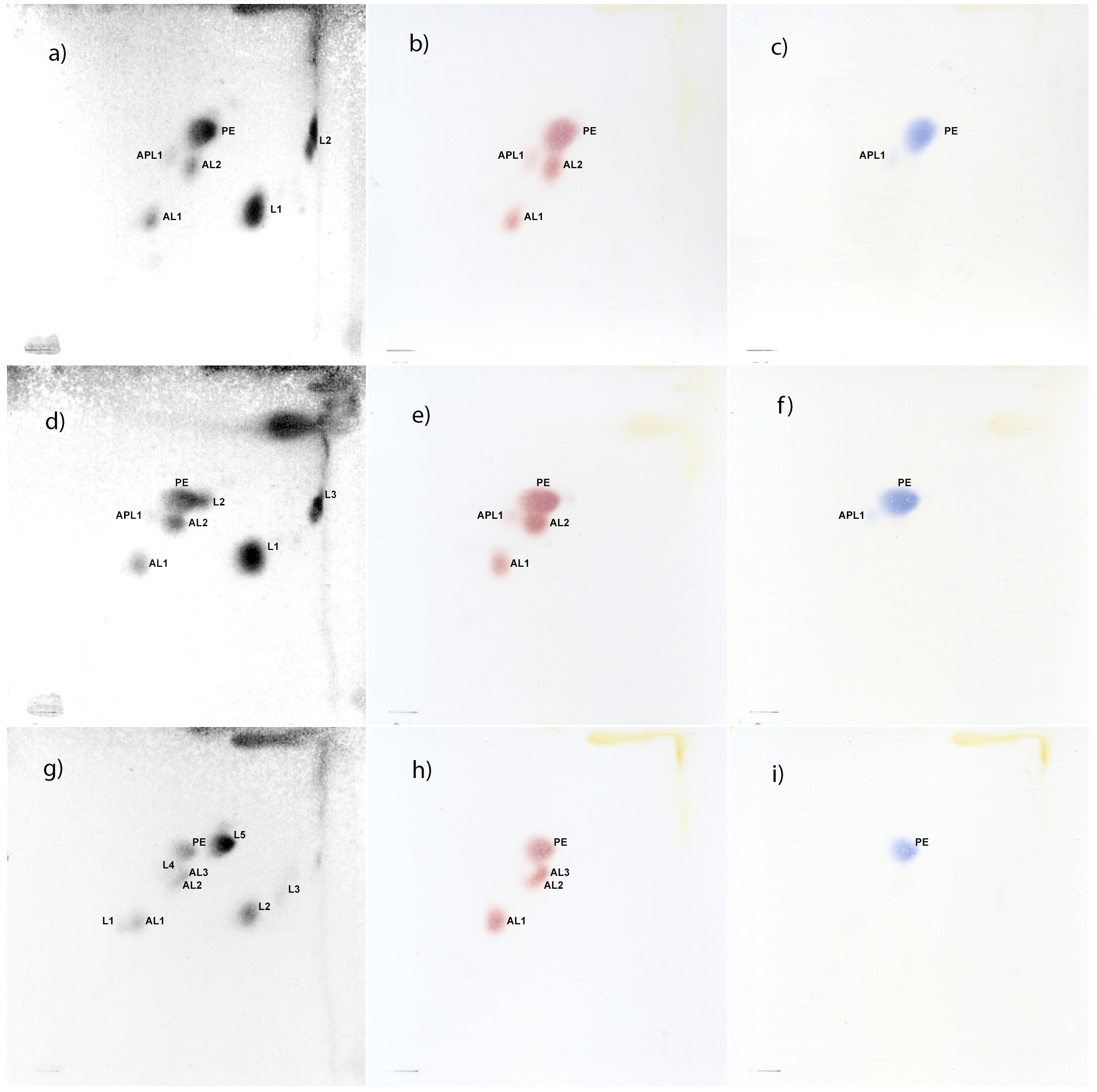


**S2 Fig.** Two-dimensional thin-layer chromatograms of polar lipids of strains: (a, b, c) KMM 9724^T^; (d, e, f) KMM 9713; (g, h, i) *Empedobacter tilapiae* KCTC 62904^T^. (a, d, g), non-specific detection of lipids prepared with 10% H_2_SO_4_ in methanol; (b, e, h), stained with ninhydrin; (c, f, i), stained with molybdate reagent. Abbreviations: PE, phosphatidylethanolamine; APL1, an unidentified aminophospholipid; AL1, AL2, AL3, unidentified aminolipids; L1, L2, L3, L4, L5, unidentified lipids.
