# Supplementary material for: Description and genome-wide analysis of Profundicola chukchiensis gen. nov., sp. nov., marine bacteria isolated from bottom sediments of the Chukchi Sea
Source: PLoS One. 2023 Jul 26;18(7):e0287346. doi: 10.1371/journal.pone.0287346 (PMC10370774; doi:10.1371/journal.pone.0287346)
Supplement: S2 File — (PDF) [file pone.0287346.s004.pdf]

G.B. Elyakov Pacific Institute of Bioorganic Chemistry
Far Eastern Branch, Russian Academy of Sciences

159 100-let Vladivostoku Prospect, Vladivostok, 690022, Russia. ☎ 7(423) 231-14-30; fax: 7(423) 231-40-50

Collection of Marine Microorganisms

CERTIFICATE of DEPOSIT and AVAILIBILITY

This is to certify that the type strain of “*Profundicola chukchiensis*” gen. nov., sp. nov. KMM 9724^{T} has been deposited and is maintained in the Collection of Marine Microorganisms (KMM), G.B. Elyakov Pacific Institute of Bioorganic Chemistry, Far-Eastern Branch, Russian Academy of Sciences. The type strain of “*Profundicola chukchiensis*” gen. nov., sp. nov. KMM 9724^{T} will be available to the public after publication of the manuscript that describes this species.

Signature__________

Name: Mikhailov V.V., Ph.D., D. Sc.
Professor of Microbiology
Curator of Collection of Marine Microorganisms
G.B. Elyakov Pacific Institute of Bioorganic Chemistry,
Far-Eastern Branch, Russian Academy of Sciences,
Prospect 100-let Vladivostoku, 159,
690022 Vladivostok,
Russia

Place and date: Vladivostok, October 7, 2022
