# Supplementary material for: Description and genome-wide analysis of Profundicola chukchiensis gen. nov., sp. nov., marine bacteria isolated from bottom sediments of the Chukchi Sea
Source: PLoS One. 2023 Jul 26;18(7):e0287346. doi: 10.1371/journal.pone.0287346 (PMC10370774; doi:10.1371/journal.pone.0287346)
Supplement: S3 File — (PDF) [file pone.0287346.s005.pdf]

KOREAN Agricultural Culture Collection (KACC)
Agricultural Microbiology Division
National Institute of Agricultural Science
166 Nongsaengmyeong-ro, Iseo-myeon, Wanju-gun
Jeollabuk-do 55365, Republic of Korea

Phone : +82-63-238-3024
Fax : +82-63-238-3845
E-mail : swkwon1203@korea.kr
Homepage : http://genebank.rda.go.kr/

Certificate of Deposition and Availability of a Microorganism

The strain ***Profundicola chukchiensis* KMM 9724** has been deposited in the Korean Agricultural Culture Collection (KACC) under the number of

KACC 22806

This strain will be available to the public without restriction after publication by author(s) of the scientific paper.

S. W. Kwon

24 July 2022
