# Supplementary material for: Description and genome-wide analysis of Profundicola chukchiensis gen. nov., sp. nov., marine bacteria isolated from bottom sediments of the Chukchi Sea
Source: PLoS One. 2023 Jul 26;18(7):e0287346. doi: 10.1371/journal.pone.0287346 (PMC10370774; doi:10.1371/journal.pone.0287346)
Supplement: Attachment — Submitted filename: Response to Reviewers.doc [file pone.0287346.s006.doc]

**RESPONSE TO REVIEWERS**

We thank the reviewers for their careful work with our manuscript. Please find below our responses to the reviewer’s comments:

***Responses for Reviewer #1:*** Thank you very much for reviewing our manuscript. We appreciate your valuable comments and suggestions. We have revised our manuscript thoroughly in accordance with your comments.

*Comments:* A new genus *Profundicola chukchiensis* gen. nov., sp. nov., was described based on genome taxonomy. Only bifurcating tree reconstruction is unlikely to show monophyletic association to the other related taxa. Authors should perform splits decomposition analysis to clearly show the monophyly of the strains described here.

Major.

1. 16S rRNA phylogeny must be shown using ML after model tests.

*Response:* Thank you for your important comment. We have made changes as recommended. Please, see revised figure 1 (**Line 280**).

2. MLSA using splits tree network must be performed.

*Response:* According to your recommendation we have performed MLSA to clarify the monophyly of the novel strains. The MLSA was conducted using concatenated sequences of the five housekeeping genes; 16S rRNA, atpD, gyrB, recA, and rpoB, which were retrieved from whole genome sequences. We have added the results (**Lines 274-276, S1 File**).

Minors

1. title was not corresponded between in manuscript and submission system.

2. L8. please put and before the last author’s name.

3. L27 and etc. please correct the font "K"MM p724T.

4. L32 and etc. one digit for similarity, ANI, AAI, and DDH should be used.

5. L54. m not meter.

6. L107. seawater. (L109)

7. L141. 16S rRNA gene sequencing.

8. L282. non-motile.

9. Fig. 4. general title should be added.

10. Table 1. capitalized firts letter of feature. no vertical and horizontal sublines.

*Response:* Thank you for your comments. We have made changes as per suggested. Please, look for them in the 'Revised Manuscript with Track Changes'.

***Responses for Reviewer #2:*** Thank you very much for reviewing our manuscript. We appreciate your valuable suggestions. We have revised our manuscript in accordance with your comments.

*Comments:* This manuscript describes the isolation of two Gram-negative bacterial strains, which based on 16S rRNA gene sequence analysis could be assigned as members of the *Weeksellaceae* family. Further analyses provided evidence for the classification of the strains as members of a novel genus, with the two strains described representing a novel species within this genus. The authors provided a report on the extensive analyses of the strains and their associated genomic features, further providing evidence for the proposed classification.

However, there are some aspects the authors need to consider/address:

1. **Lines 67-70**: The list of the members belonging to the family *Weeksellaceae* needs to be updated – please see LPSN.dsmz.de for a more complete list.

*Response:* Thank you for your comments. We have updated the list of the members belonging to the family *Weeksellaceae* as per suggested. Please, see **Lines 80-82.**

2. **Lines 101, 106, 362:** For any % solution reported, please indicate what it represents, i.e., v/v or w/v, etc.

*Response:* Thank you for your comments. We have made changes as per suggested.

3. **Line 110:** Please indicate whether an artificial sea water (ASW) control was setup for the API analyses, especially since the strains were suspended in ASW as indicated.

*Response:* Yes, indeed, all controls were made as required.

4. **Line 148**: BLAST analysis and EzBioCloud analysis were performed in 2021. Please indicate whether the same results were obtained during a more recent (2023) search? Sequence information in databases is growing at a phenomenal rate – a more recent analysis would be more accurate.

*Response:* We agree with your comment. We have checked again and corrected the access date. Please, see the **Lines 167**.

5. **Lines 187-189**: dbCAN2 has been updated to dbCAN3 – it would be of value to re-run the genome sequences on the updated version in order to confirm whether the outputs are the same or whether the updated version changes the results presented. dbCAN3 output files should also ideally be downloaded and made available via a data repository.

*Response*: Thank you so much for your important comment. The genome sequences were annotated via dbCAN3, and output files are presented as tables in supplementary (**S1 File**). We have added this information to the text of the manuscript. Please, see **Lines 243-246.** We have to say that predicted CAZyme quantities for both genomes obtained by versions 2 and 3 differed very slightly.

6. **Lines 195-196**: Please provide the scripts used for the analyses in R – either as supplementary material or made accessible via a data repository.

*Response*: Thank you for your comment. We used R only for the visualization purposes. We analyzed output files from ANI/AAI-Matrix, TYGS platform, eggNOG-mapper v2, and dbCAN2 meta servers, and corresponding tables were imported in RStudio to obtain heatmaps by the pheatmap package according to manual.

7. **Line 200**: Even though the genome sequence accession numbers are provided, neither provide a hit in GenBank. The same applies to ‘OP604014’. Only ‘LC379507’ returns a result in GenBank for a Flavobacteriaciae bacterium Ch26. Please update the GenBank entry to correspond to what is being reported in the manuscript and ensure that the data linked to the other accession numbers are released. Not being able to access the genomes made it difficult to assess whether what was reported in the manuscript is reproducible.

*Response*: We apologize for that. Now, we have released all the sequences reported in the manuscript.

8. Minor: Please correct the spelling of ‘source’ in **line 349**, and ‘designed’ in **line 26** should be ‘designated’.

*Response*: We apologize sincerely for these typo mistakes. They are rewritten in the revised manuscript.

We hope that the revised manuscript has been improved and is suitable for publication in “PLOS ONE”.

Sincerely yours,

Marina P. Isaeva,

PhD, Associate Professor,

The Head of the Laboratory of Marine Biochemistry

Elyakov Pacific Institute of Bioorganic Chemistry FEB RAS,

prospekt 100-let Vladivostoku, 159

Vladivostok, 690022

Russia

e-mail: issaeva@gmail.com
